# Inducible secretion of LIGHT by engineered probiotics enables localized cytokine therapy and robust antitumor immunity

**DOI:** 10.1039/d5ra09644h

**Published:** 2026-05-18

**Authors:** Xinyu Li, Shuaijie Ding, Biao Yang, Yi Wang, Wei Xie

**Affiliations:** a School of Food and Nutrition, Joint Research Center for Food Nutrition and Health of IHM, Anhui Agricultural University Hefei Anhui 230036 China; b Institute of Health and Medicine (IHM), Hefei Comprehensive National Science Center Hefei Anhui 230000 China xiew@ihm.ac.cn; c Department of Immunology, School of Basic Medical Sciences, Center for Big Data and Population Health of IHM, Anhui Medical University Hefei Anhui 230032 China

## Abstract

Triple-negative breast cancer (TNBC), exemplified by the 4T1 model, exhibits a highly immunosuppressive tumor microenvironment (TME) and strong metastatic potential, resulting in poor responses to current immunotherapies. TNFSF14 (LIGHT) is a potent immunostimulatory cytokine capable of remodeling the TME through the HVEM and LTβR signaling. However, its systemic administration is limited by dose-dependent toxicity. Here, we developed a tumor microenvironment-responsive engineered *E. coli* system for targeted LIGHT delivery. LIGHT expression was controlled by a lactic acid-inducible promoter and fused with pelB for periplasmic secretion, ensuring selective activation within lactic acid-rich tumor cores. In BALB/c mice bearing 4T1 subcutaneous tumors and experimental lung metastases, intravenously administered bacteria were evaluated for biodistribution, antitumor efficacy, and immune modulation. The engineered strain selectively colonized tumors, achieving strong intratumoral LIGHT expression with minimal systemic exposure. Compared with vector controls, LIGHT-expressing bacteria significantly suppressed primary tumor growth and markedly reduced lung metastatic lesions. Mechanistically, this treatment increased intratumoral CD8^+^ T-cell infiltration, enhanced dendritic cell maturation, and shifted the TME toward an immune-activated state. Thus, this lactic acid-responsive bacterial platform enables safe, localized cytokine delivery and represents a promising therapeutic strategy for refractory TNBC.

## Abstract 2

Lactic acid-responsive engineered probiotics selectively colonize TNBC tumors. By secreting LIGHT, they remodel the immunosuppressive microenvironment and inhibit tumor growth, which is a promising targeted treatment approach.

## Graphical abstract

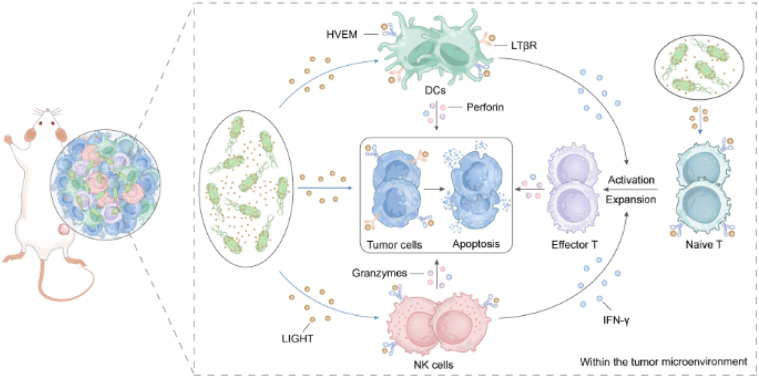

## 1. Introduction

Cancer is the leading cause of death worldwide, and breast cancer stands out as the most commonly diagnosed malignancy among women, posing a significant public health burden.^1^ Within breast cancer subtypes, TNBC is notably aggressive, characterized by the absence of estrogen receptor (ER), progesterone receptor (PR), and human epidermal growth factor receptor 2 (HER2) expression.^2,3^ This lack of actionable molecular targets renders TNBC unresponsive to endocrine or HER2-targeted therapies, leaving patients with limited treatment options.^4,5^ Compounding this challenge, TNBC exhibits a highly immunosuppressive TME, featuring sparse cytotoxic T-cell infiltration, abundant regulatory T cells, M2-polarized macrophages and strong metastatic potential, often leading to early relapse and poor overall survival.^6,7^ While immune checkpoint blockade (ICB) has transformed the treatment of some solid tumors, its efficacy in TNBC remains modest, with only a small subset of patients achieving durable responses.^8,9^ This clinical gap underscores an urgent need for innovative therapeutic strategies that can reprogram the immunosuppressive TME of TNBC and elicit robust, sustained antitumor immunity.

In recent decades, bacteria-mediated cancer therapy has emerged as a promising approach to address these unmet needs, leveraging the unique ability of certain attenuated bacterial strains to selectively colonize hypoxic, necrotic tumor cores-microenvironments where conventional drugs and immune cells frequently fail to penetrate.^10,11^*Escherichia coli* Nissle 1917 (EcN), a well-characterized probiotic,^12^ is particularly well-suited for this role: it boasts a proven safety profile in humans, exhibits natural tropism for tumor tissues, and is genetically tractable, enabling its engineering to produce or deliver therapeutic molecules directly within the TME.^13,14^ Preclinical studies have shown that EcN can disrupt the TME by triggering local inflammatory responses, activating innate immune cells (*e.g.*, macrophages and dendritic cells), and enhancing T-cell infiltration.^15^ However, as a monotherapy, EcN often fails to fully reverse the deep-seated immunosuppression in aggressive tumors like TNBC, highlighting the need to combine engineered bacteria with potent immunomodulatory agents to achieve synergistic antitumor effects.^16,17^

LIGHT, a member of the tumor necrosis factor (TNF) superfamily, has gained attention as a potent immunomodulatory molecule suitable for next-generation combination immunotherapies.^18–20^ Through engagement with its receptors HVEM (TNFRSF14) and LTβR, LIGHT orchestrates a broad spectrum of immune-activating processes.^21^ It drives dendritic cell maturation by upregulating co-stimulatory molecules (CD80 and CD86), enhances the priming, activation, and expansion of CD8^+^ cytotoxic T cells, augments NK cell activity, and promotes macrophage polarization toward a pro-inflammatory M1 phenotype-collectively contributing to the reversal of tumor-induced immunosuppression.^22–24^ Despite its therapeutic potential, systemic administration of LIGHT is hindered by dose-dependent toxicity and rapid serum clearance, which narrow its therapeutic window and limit clinical applicability.^25,26^ Realizing the full therapeutic potential of LIGHT, therefore, requires a delivery strategy capable of precise spatiotemporal control, ensuring that LIGHT expression and secretion are tightly confined to the TME.

**Fig. 1:**
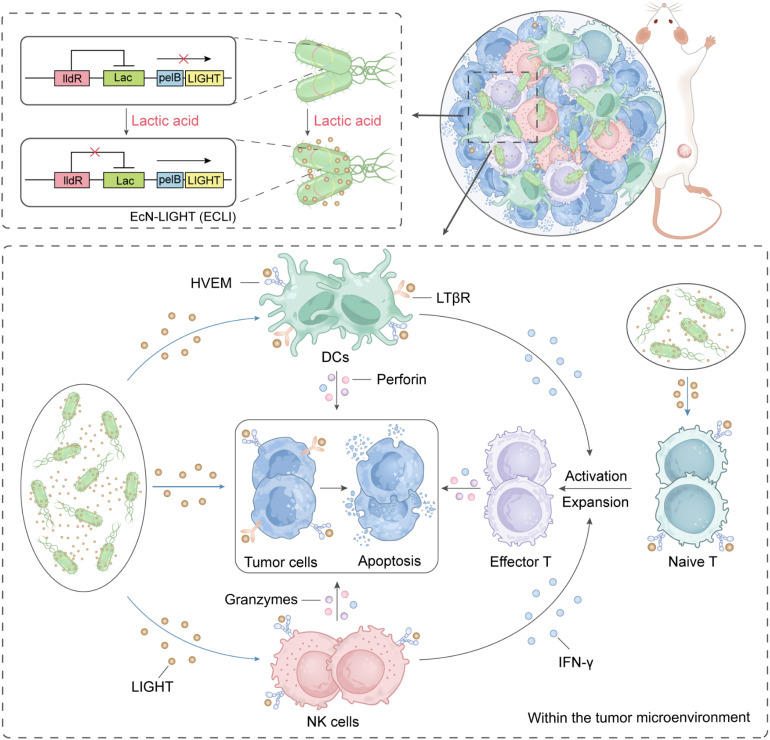
Schematic of a bacteria-mediated cancer immunotherapy model. The expression of LIGHT was induced in engineered bacteria that are responsive to lactic acid by fusing the pelB upstream of LIGHT. In the presence of lactic acid, this drives the engineered bacteria to secrete LIGHT outside the bacterial cell. This leads to the activation of dendritic cells, which in turn stimulate the effector T cells and NK cells. These activated immune cells ultimately target and induce the death of tumor cells.

A defining hallmark of the TNBC TME that enables such targeted delivery is its elevated lactic acid concentration, a byproduct of the Warburg effect (aerobic glycolysis).^27^ Aggressive tumors like TNBC rely heavily on aerobic glycolysis to meet their high metabolic demands, leading to lactic acid accumulation in the TME. This tumor-specific lactic acid enrichment makes it an ideal endogenous cue for TME-responsive gene regulation.^16,28,29^ In this study, we engineered a novel therapeutic platform, EcN-LIGHT, produced by lactic acid (ECLI), by modifying EcN to secrete LIGHT under the control of the lactic acid-inducible LldPRD promoter, a genetic circuit that ensures LIGHT expression is activated only in the presence of high lactic acid levels.^30,31^ Additionally, we fused LIGHT with the PelB signal peptide to facilitate efficient periplasmic secretion, ensuring that bioactive LIGHT is released directly into the TME.^32^ Our investigations focused on assessing the ability of ECLI to inhibit primary tumor growth, suppress distant metastasis, prolong animal survival, and remodel the TME. By validating this lactic acid-responsive, bacteria-mediated LIGHT delivery system, we aim to provide a safe and effective therapeutic strategy for TNBC and other immunologically “cold” solid tumors, addressing a critical unmet clinical need (Fig. 1).

## 2. Experimental section

### 2.1. Materials

EcN was purchased from Baosai Biotech Company. Lactic acid (catalog no. HY-B2227, CAS no. 50-21-5) was purchased from MCE. APC/Cy7 anti-mouse CD45, FITC anti-mouse CD3, PE anti-mouse CD80, APC anti-mouse CD86, APC anti-mouse CD8, PE anti-mouse CD49b, PE/Cy7 anti-mouse CD86, FITC anti-mouse F4/80 were purchased from Biolegend. Anti-rabbit antibody 647 (A31573) was purchased from Invitrogen. Penicillin, streptomycin, LB basal medium, nonfat powdered milk, BeyoECL Plus, protein extraction kit, ampicillin, chloramphenicol, anti-FLAG tag-HRP were purchased from Beyotime Biological Company. DMEM and RPMI-1640 medium were purchased from Ketu Biotechnology. FBS was purchased from Gibco. Plasmid extraction kit and DNA recovery kit were purchased from Tiangen Biotech. ELISA kits were purchased from Boster Biological Technology. All chemicals were of analytical reagent grade, and deionized water was used in all experiments.

### 2.2. Plasmids and bacterial transformation

EcN was cultured in LB broth (Solarbio) with vigorous shaking at 37 °C. EcN was then kept in a bacterial cryopreservation solution (50% LB broth, 25% water, 25% glycerol) at −80 °C for further experiments.

The lactic acid-responsive GFP plasmid was synthesized from the whole sequence by Genewiz Biotech Company. The LIGHT fragment was amplified by PCR, and the target gene fragment was isolated by agarose gel electrophoresis. DNA recovery was performed on the target gene fragment, and the vector fragment was cloned in the same way. The vector fragment contained a 15 bp homologous arm sequence. Seamless cloning was then used to ligate LIGHT to the vector fragment and transform it into DH5α. After an overnight culture, monoclonal clones were selected and sent for Sanger sequencing verification. Plasmid electroporation (1.8 kV, 5 ms) was performed to introduce the plasmid into the EcN host strain, and positive clones of the transformed strains were screened on ampicillin-containing LB medium.

### 2.3. GFP fluorescence detection

Lactic acid-responsive-GFP bacteria were inoculated into LB medium containing ampicillin. When the optical density of the bacteria reaches 0.6, different concentrations of lactic acid are gradually added to promote the expression of GFP. The bacteria were further cultured for 24 hours and then collected. Fluorescence microscopy was used to observe the expression of GFP.

### 2.4. ELISA and western blot assays for protein expression

Total bacterial proteins were collected from supernatants obtained after treatment with different concentrations of lactic acid. Briefly, after 12 hours of induction, cultures were centrifuged at 4 °C and 8000 rpm for 15 minutes to remove bacterial cell pellets. The supernatant was filtered through a 0.22 µm filter to remove residual bacteria and then concentrated using TCA, followed by protein sample preparation and analysis. ELISA was used to quantify the concentration of labeled proteins in the supernatants. Proteins were separated using SDS-PAGE and transferred onto PVDF. The PVDF membranes were then coated with a 5% BSA solution in TBST and incubated with anti-Flag tag-HRP at 4 °C overnight. Subsequently, the PVDF membranes were washed with TBST and developed using ECL for imaging.

### 2.5. Cancer cell lines and animals

4T1 (mouse breast cancer cells) and NK-92 were purchased from the American Type Culture Collection (ATCC, USA). 4T1 cells were cultured in DMEM medium. The medium contained 10% fetal bovine serum (FBS) and 1% penicillin–streptomycin. NK-92 cells were cultured in an MEMα basal medium containing 12.5% heat-inactivated horse serum, 12.5% FBS, 1% PBS, 0.2 mM inositol, 0.1 mM β-Mer, 0.02 mM folic acid, and 100–200 U mL^−1^ recombinant IL-2. All cells were cultured in a 37 °C incubator under a 5% carbon dioxide atmosphere.

Female BALB/c mice (6–8 weeks old) were purchased from Jicui Pharmachem Biotechnology Company. Place the mice in ventilated cages, with 5 mice in each cage. Control the temperature (21 ± 1 °C) and humidity (40–70%) of the cages. Regularly implement a 12-hour : 12-hour light-dark cycle. Food and water were provided ad libitum. All animal experiments were conducted in accordance with relevant national regulations, animal welfare guidelines and policies of the Institute of Health and Medicine, Hefei Comprehensive National Science Center (IHM), and were approved by the Institutional Animal Care and Use Committee (IACUC) of IHM.

### 2.6. T-cell proliferation

Pure T cells were isolated from the spleen of mice and resuspended in PBS. They were then added with a final concentration of 2.5 µM CFSE fluorescent dye and incubated at 37 °C in the dark for 10–20 minutes. To terminate the staining, five volumes of serum-containing complete medium were added immediately, and the cells were washed twice by centrifugation to remove unbound dye completely. The CFSE-labeled T cells were resuspended and inoculated into 96-well plates. Different bacterial supernatants were added to treat the T cells. After culturing the cells at 37 °C in a 5%-CO_2_ incubator for 3 days, the cells were collected, and the attenuation of CFSE fluorescence intensity was detected using a flow cytometer. The proliferation ratio and division generation of T cells were analyzed by FlowJo, and the level of cytokines was detected by ELISA.

### 2.7. Obtaining bone-marrow-derived dendritic cells (BMDCs)

First, the alcohol-sterilized tibia and femur were rinsed with PBS and then filtered through a 200-mesh sterile filter to obtain BMDCs. The filtrate was centrifuged at 1800 rpm for 5 minutes, the cells were resuspended in 2 mL of red blood cell lysis buffer and incubated for 2 minutes. After another centrifugation, the BMDCs were resuspended in RPMI-1640 medium supplemented with IL-4 (10 ng mL^−1^) and GM-CSF (10 ng mL^−1^). The cells were cultured for 6 days to obtain immature dendritic cells. On day 3, the culture was replenished with equal volumes of IL-4 and GM-CSF. Supernatants from different engineered bacteria were added and incubated for 24 hours, and then flow cytometry was performed to detect CD80 and CD86 expression on BMDCs. Supernatants from lactic acid-induced engineered bacteria were treated with LIGHT neutralizing antibodies and incubated for 30 minutes, and the incubated product was then used to stimulate BMDCs for 24 hours, and the expression of CD80 and CD86 was detected by flow cytometry.

### 2.8. Analysis of NK-92 cells

NK-92 cells were seeded in 12-well plates and treated with LB medium, supernatants from engineered bacteria or recombinant LIGHT. After 24 hours of treatment, the cells were collected for analysis.

### 2.9. Tumor model

For the subcutaneous tumor model, 4T1 cells (5 × 10^6^) were injected subcutaneously (in 100 µL PBS) into BALB/c mice, which were anesthetized with 2% isoflurane prior to injection. Tumor volume reached 100–120 mm^3^ at 7–8 days post-injection, at which point mice received the indicated types of engineered bacteria *via* intratumoral injection, 5 × 10^6^ colony-forming units (c.f.u.). When administering the engineered bacteria to the LIGHT group and the ECLI group, the recombinant protein was injected into the tumor. The concentration of recombinant LIGHT used was 1 µg mL^−1^, administered once every 3 days, with a single dose of 5 µg per animal.

The tumor volume (mm^3^) was measured every two days. The calculation formula was: (*axb*^2^)/2, where *a* is the height and *b* is the width. Mice with a tumor volume of 1500 mm^3^ or more were euthanized as per the requirements of the ethics committee. Mice that exhibited symptoms of pain, discomfort, or distress were immediately euthanized. Most of the data analyses and experiments were performed in a blinded manner to prevent bias.

### 2.10. Cytokine detection by ELISA

The concentrations of the cytokines IFN-γ, IL-1β, IL-2, and TNF-α were measured using ELISA kits.

### 2.11. Flow cytometry

Solid tumors were excised from mice after bacterial treatment and soaked in 2 mL of isolation buffer (RPMI-1640 medium containing 5% FBS, 1% l-glutamine, 1% penicillin–streptomycin, and 10 mM HEPES). After mechanically homogenizing the tumor tissue, the cell suspension was treated with 1 mg mL^−1^ collagenase IV and 50 µg mL^−1^ Nase I, and incubated at 37 °C for 30 minutes. Next, 2 mL of each sample were taken and mixed with an equal amount of red blood cell lysis solution. The mixture was incubated at 37 °C for 4 minutes to remove the red blood cells. Then, the cells were filtered through a 40 µm filter membrane. Finally, the cells were washed sequentially with FACS buffer (2% FBS in DPBS). Cells were then incubated for 10 min at room temperature with Fc blocking buffer (BioLegend) to prevent non-specific antibody binding. According to the instructions provided by the antibody manufacturer, the antibody coupled with a specific fluorescent dye should be stained on ice for 30 minutes. Data were acquired on a FACS Canto flow cytometer and analyzed using FlowJo software. A minimum of 10^6^ cellular events were recorded per sample.

### 2.12. Histological analysis

Extract the isolated tissue of the mouse tumor, fix it in 4% paraformaldehyde for 24 h, embed the tumor tissue in paraffin, make sections, and conduct tissue immunofluorescence and H&E staining. Observe under a light microscope. According to the previously described standards, efforts are made to minimize observer bias.

### 2.13. *In vivo* toxicity assessment

Ten female BALB/c mice (6–8 weeks old) were divided into two groups (five mice per group) and intratumoral (i.t.) injections of PBS or ECLI on days 3, 5, and 7. The mice were euthanized on day 10, and blood samples were collected and sent to Anhui KETU Biotechnology Co., Ltd. for routine hematology and blood biochemistry analysis. Major organs were also collected for histopathologic evaluation by H&E staining.

### 2.14. Statistics

Data are expressed as the mean ± SEM. Statistical differences between control and experimental groups were analyzed by one-way ANOVA followed by Tukey's *post hoc* test. Paired samples were compared *via* a unpaired *t*-test. No data were excluded from the analyses. Statistical significance is indicated as *P < 0.05, **P < 0.01, ***P < 0.001, and ****P < 0.0001.

## 3. Results

### 3.1. Construction and characterization of the lactic acid-responsive LIGHT-secreting engineered bacterium

**Fig. 2:**
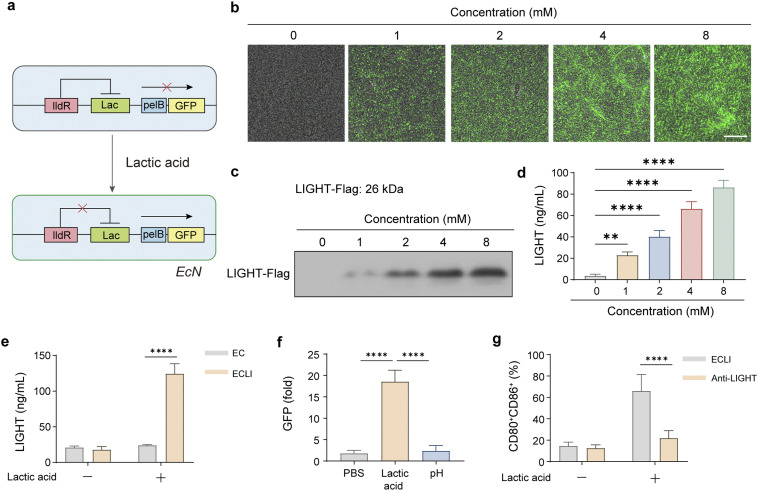
Secretion of LIGHT produced by lactic acid-induced engineered bacteria and their functional validation. (a) Genetic map of the lactic acid-pelB-LIGHT plasmid. (b) Representative images of fluorescence microscopy, showing the dose-dependent, lactic acid-induced expression of GFP; scale bar = 10 µm. (c and d) Western blot and ELISA analyses, quantifying intracellular *versus* extracellular LIGHT after induction. (e) LIGHT levels secreted by EC and ECLI bacteria following lactic acid induction. (f) GFP expression in bacteria exposed to buffer and lactic acid at the same pH level. (g) Flow cytometric analysis of the proportion of mature CD80^+^CD86^+^ cells following treatment with the lactic acid-induced bacterial LIGHT in the presence or absence of LIGHT-neutralizing antibodies. Data are presented as mean values ± SEM (*n* = 3 biologically independent samples for (c) and (d)).

To achieve tumor microenvironment-responsive cytokine delivery, we constructed an engineered *E. coli* Nissle 1917 (EcN) strain expressing pelB-fused murine LIGHT under the control of a lactic acid-inducible promoter (LldPRD system). The lactic acid sensor LldR and its cognate promoter were placed upstream of the pelB-LIGHT cassette (Fig. 2a). A control plasmid expressing GFP under the same promoter was also generated (Sup Fig. 1).

To validate promoter responsiveness, EcN-GFP was cultured with increasing concentrations of sodium lactic acid (0–8 mM), which reflects physiological tumor-lactic acid levels. Fluorescence microscopy revealed dose-dependent activation, consistent with the known induction range of the LldPRD system (Fig. 2b). Negligible basal signal confirmed tight promoter repression in the absence of lactic acid. Subsequently, the coding sequence of GFP was substituted with that of LIGHT, and the recombinant engineered bacteria were induced using lactic acid as the inducer. The engineered bacteria showed no effect on growth (SI Fig. 2). Western blot analysis of EcN-LIGHT demonstrated efficient induction of LIGHT expression upon lactic acid stimulation, with most protein detected in the culture supernatant rather than cell lysates, indicating effective pelB-mediated secretion (Fig. 2c). Quantification by ELISA showed a strong dose-response relationship, with LIGHT secretion increasing proportionally to lactic acid concentration (Fig. 2d).

To confirm that bacterial protein expression was driven by the lactic acid-induced plasmid, we used lactic acid to induce EC and ECLI separately. The results shown in Fig. 2e indicate that only the group expressing the plasmid and treated with lactic acid exhibited a significant increase in LIGHT. Next, to verify the specificity of lactic acid-induced expression, we adjusted the pH to the same level and assessed GFP expression. The results shown in Fig. 2f indicate that only the bacteria in the lactic acid-treated group exhibited a significant increase in GFP expression. Since LIGHT can induce the activation or maturation of various immune cells, we conducted validation experiments using dendritic cells (DCs). The results in Fig. 2g show that DCs in the group treated with LIGHT-neutralizing antibodies exhibited significantly reduced maturation. This indicates that the maturation of DCs induced by the engineered bacteria is caused by LIGHT secretion, and that this effect can be attenuated by neutralizing antibodies. These data confirm that the lactic acid-responsive system enables a strict on/off switch, pelB ensures efficient secretion, and bacterially produced LIGHT retains full antitumor bioactivity.

### 3.2. LIGHT produced by engineered bacteria enhances T-cell proliferation and effector function

**Fig. 3:**
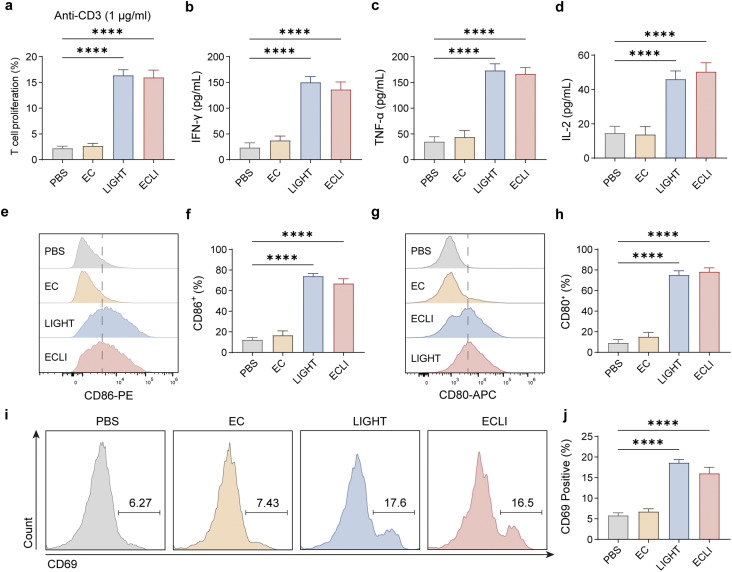
LIGHT promotes the proliferation and activation of immune cells. (a) Proliferation of CFSE-labeled T cells co-incubated with supernatants from lactic acid-induced engineered bacteria, as detected by flow cytometry. (b–d) ELISA quantification of cytokine levels in T-cells after co-incubation with bacterial supernatants. (e–h) Flow cytometry analysis of BMDC maturation: positive rates of CD80 (e) and CD86 (g) after treatment with the supernatant of engineered bacteria, with corresponding statistical graphs. (h and i) Flow cytometry analysis and statistics of NK-92 cell activation after bacterial supernatant treatment. Data are presented as mean values ± SEM (*n* = 3 biologically independent samples for (a)–(d), (f), (h), and (j)).

In order to determine whether LIGHT secreted by the engineered bacteria can stimulate adaptive immune responses, CFSE-labeled mouse splenic T cells were cultured separately with PBS, EcN (EC), the supernatant of ECLI, and the recombinant protein of LIGHT (100 ng mL^−1^).^33^ Flow cytometry showed that CD8^+^ T cells exposed to LIGHT underwent robust proliferation, as indicated by a marked decrease in CFSE intensity compared with control groups (Fig. 3a). Correspondingly, ELISA measurements revealed significantly elevated levels of IFN-γ, TNF-α, and IL-2 in the EcN-LIGHT group, indicating a shift toward a cytotoxic effector phenotype (Fig. 3b–d). Supernatants from non-induced EcN produced little or no cytokine release, further confirming the tight inducibility of the system. These data demonstrate that lactic acid-induced LIGHT expression from engineered EcN drives both the proliferation and functional polarization of CD8^+^ T cells.^34^

### 3.3. LIGHT induces dendritic cell maturation and NK-cell activation

We next assessed whether LIGHT modulates innate immune activation. BMDCs treated with supernatants from lactic acid-induced LIGHT-expressing bacteria exhibited strong upregulation of the maturation markers CD80 and CD86 (Fig. 3e–h). This indicates efficient initiation of antigen-presenting activity. Similarly, NK-92 cells treated with LIGHT displayed significantly elevated CD69 expression, demonstrating activation of innate cytotoxic responses (Fig. 3i and j). Together, these data show that bacterially produced LIGHT enhances both innate and adaptive immune responses essential for antitumor activity.

### 3.4. Selective intratumoral colonization and persistence of engineered bacteria

**Fig. 4:**
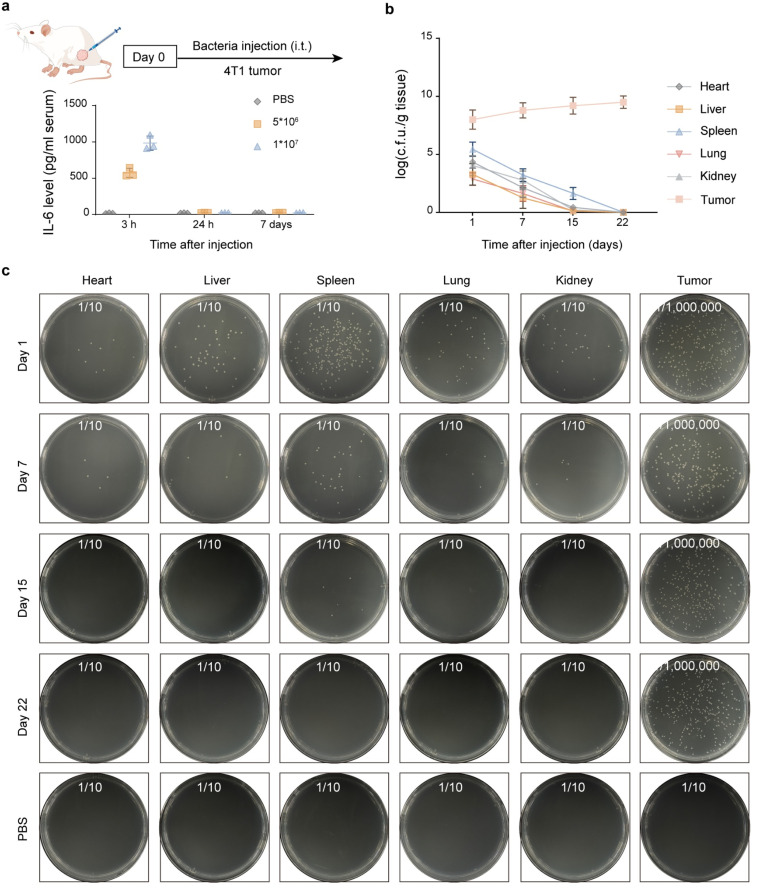
*In vivo* biodistribution and persistence of engineered bacteria after intratumoral injection. (a) Serum IL-6 concentrations at 3 h, 24 h, and 7 days following the intratumoral administration of different bacterial doses (5 × 10^6^ or 1 × 10^7^ CFU). Transient early IL-6 elevation subsides by 24 h, indicating self-limiting inflammation. (b) Quantitative measurement of bacterial load in major organs and different tumor types at various time points following injection. (c) Representative colony formation from organ homogenates at corresponding time points. Persistent bacterial growth was observed only in tumor tissues up to day 22; no colonies were detected in PBS controls. *n* = 5 biologically independent samples for (a) and (b).

To assess *in vivo* behavior of the engineered strain, BALB/c mice bearing 4T1 tumors received a single intravenous dose of EcN-Lactic acid constructs (two inocula tested: 5 × 10^6^ and 1 × 10^7^ CFU). Bacterial biodistribution, systemic cytokine induction and organ colonization were analyzed at multiple time points.^13^ Serum IL-6 (an acute innate marker) showed a transient, dose-dependent elevation at 3 h post-injection that returned to baseline by 24 h in both dose cohorts; no persistent systemic cytokine elevation was observed at later time points (days 7), indicating the absence of a prolonged systemic inflammatory response (Fig. 4a). Quantitative culture (log CFU per gram of tissue) demonstrated rapid tumor enrichment: by day 1, tumors contained high CFU counts, whereas CFU levels in liver, spleen, lung, heart and kidney dropped precipitously (Fig. 4b). By day 15, bacteria were essentially confined to tumors, and by day 22, non-tumor tissues were culture-negative in most animals (Fig. 4b and c). Representative colony plates (serial dilutions shown) confirmed this pattern: tumor homogenates produced dense colonies even at 10^6^-fold dilutions, while plates from other organs had few or no colonies at the same dilutions and became sterile by days 15–22 (Fig. 4c). PBS-treated controls showed no colonies in any organ at any time point.

Before the application of engineered probiotics, the intrinsic safety of EcN was carefully evaluated in healthy BALB/c mice. Hematoxylin and eosin (H&E) staining of major organs (heart, liver, spleen, lung, and kidney) revealed comparable histological features between the PBS and ECLI groups, with no detectable tissue injury, necrosis, or inflammatory infiltration, and intact structures of hepatic lobules, cardiac muscle fibers, splenic pulps, alveolar tissues, and renal glomeruli/tubules (SI Fig. 3). Serum biochemical indices were similar between ECLI-treated and control mice, indicating preserved liver function and metabolic stability; all hematological parameters were within normal physiological ranges, confirming the absence of systemic infection, excessive immune activation, anemia or hematological toxicity (SI Fig. 4 and 5). Body weight remained unchanged (SI Fig. 6). In summary, the combined results of histopathological, biochemical and hematological analyses indicate that EcN is biocompatible and does not cause detectable systemic side effects. To verify the expression of ECLI within mouse tumors, we performed intratumoral injection of ECLI and detected Flag expression in the tumor, which demonstrated that LIGHT is efficiently expressed within the tumor (SI Fig. 7a). At the same time, no significant increase was observed in serum levels (SI Fig. 7b). To verify whether the bacteria remained functional within the tumor, the EC-GFP strain was collected following intratumoral injection. Upon *in vitro* induction with lactic acid, GFP expression increased significantly, indicating that the engineered bacteria retained their function *in vivo* (SI Fig. 7c).

Together, these data indicate strong tumor tropism and durable intratumoral persistence of EcN along with rapid clearance from healthy organs. The transient IL-6 spike is consistent with an early innate immune response that is rapidly resolved, supporting an acceptable safety profile for systemic delivery in this model.

### 3.5. ECLI inhibits tumor growth and prolongs survival in 4T1 model

**Fig. 5:**
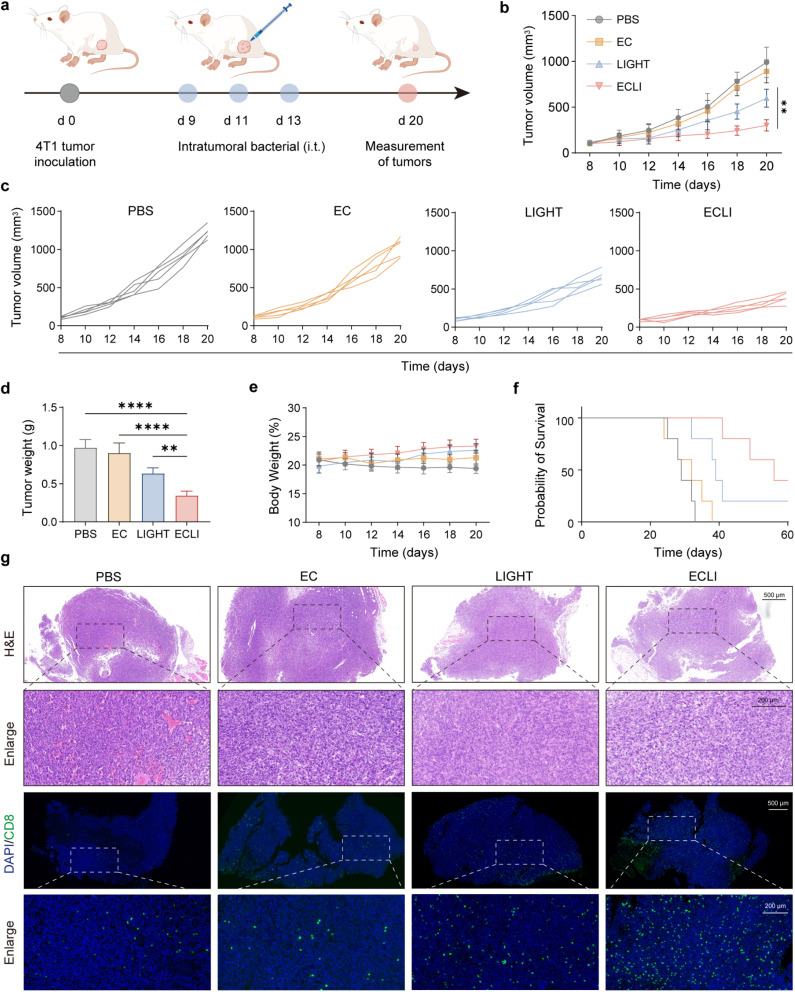
Evaluation of the antitumor efficacy of LIGHT secreted by engineered bacteria. (a) Schematic of the experimental procedure: 4T1 tumor cells were inoculated on day 0, followed by intratumoral bacterial administration on days 9, 11, and 13 and tumor assessments on day 20. (b) Tumor volume dynamics of mice in the PBS, EC, LIGHT, and ECLI groups over time. (c) Individual tumor volume growth curves for each experimental group. (d) Quantification of tumor weight on day 20. (e) Body weight changes in mice during the experiment, indicating no notable treatment-related toxicity. (f) Kaplan–Meier survival curves illustrating the survival probability of mice in different groups. (g) H&E staining (upper panels) and CD8 immunofluorescence staining (lower panels) of tumor tissues, showing histological characteristics and CD8^+^ T-cell infiltration; scale bars: 500 µm (upper) and 200 µm (lower). Data are presented as mean values ± SEM (*n* = 5 biologically independent samples for (b), (d), and (e)).

To assess the therapeutic performance of the lactic acid-responsive bacterial system, 4T1 tumors were established subcutaneously in BALB/c mice.^35,36^ Once tumors reached approximately 100 mm^3^, animals were randomly divided into four groups: (1) PBS, (2) EC, (3) recombinant murine LIGHT protein, and (4) ECLI. Intratumoral administration of bacteria was conducted on days 9, 11, and 13 (Fig. 5a), with tumor volume measurements performed every alternate day (Fig. 5b). By day 20, the PBS and EcN groups showed rapid tumor growth, with volumes exceeding 1100 mm^3^, while the recombinant LIGHT group exhibited moderate tumor growth inhibition. In sharp contrast, the ECLI group displayed significant tumor regression, maintaining volumes below 300 mm^3^ over the entire course of the study (Fig. 5c). On the 20th day, the mouse tumors were collected. The trend of the weight and volume of the tumors was consistent. The ECLI group showed excellent therapeutic efficacy (Fig. 5d). Meanwhile, we continuously analyzed the weight of the mice (Fig. 5e) and the survival rate of the mice (Fig. 5f). After ECLI treatment, the mice could significantly extend their survival period compared to the control group. Therefore, the tumor tissues were analyzed. The results of H&E staining and CD8^+^ T-cell immunofluorescence showed that the tumors in the ECLI group exhibited more T-cell infiltration. These results demonstrate that localized, inducible LIGHT expression by engineered bacteria achieves significantly stronger antitumor efficacy than direct cytokine injection.

### 3.6. ECLI induces profound remodeling of the tumor immune microenvironment and potent multidimensional antitumor immune activation

**Fig. 6:**
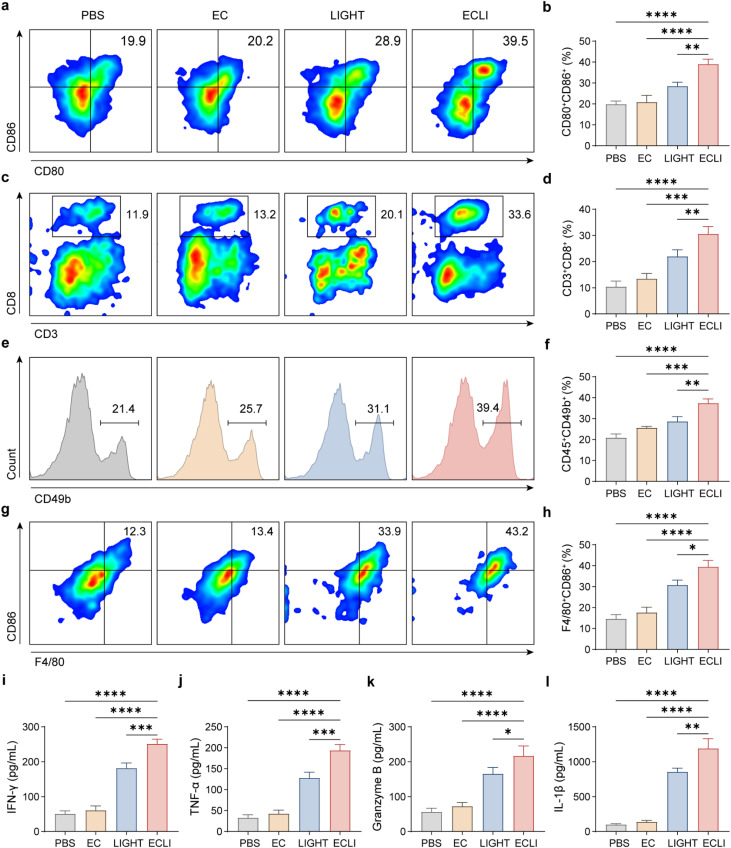
ECLI potently activates antitumor immune cells and enhances proinflammatory cytokine secretion in the tumor microenvironment. (a and b) Flow cytometry contour plots (a) and quantitative analysis (b) of CD80^+^CD86^+^ antigen-presenting cells in tumor tissues from the PBS, EC, ECLI, and LIGHT groups. (c and d) Flow cytometry contour plots (c) and quantitative analysis (d) of CD3^+^CD8^+^ cytotoxic T lymphocytes in tumor tissues across groups. (e and f) Flow cytometry histograms (e) and quantitative analysis (f) of CD49b^+^ NK cells in tumor tissues. (g and h) Flow cytometry contour plots (g) and quantitative analysis (h) of F4/80^+^CD86^+^ M1-polarized macrophages in tumor tissues. (i–l) Levels of IFN-γ (i), TNF-α (j), IL-1β (k), and granzyme B (l) in tumor tissues, measured by ELISA. Data are presented as mean values ± SEM (*n* = 5 biologically independent samples for (b), (d), (f), (h), and (i)–(l)).

Given that LIGHT exerts potent regulatory effects on both innate and adaptive immunity (as previously described), subsequent analyses are directed toward investigating the landscape of tumor-infiltrating immune cells.^31^ Specifically, flow cytometry analysis showed that the proportion of CD80^+^CD86^+^ double-positive antigen-presenting cells in the ECLI group was markedly higher than that in the PBS, EC, and LIGHT groups (Fig. 6a, b and SI Fig. 8), indicating a marked enhancement in APC activation. Concurrently, the percentage of CD3^+^CD8^+^ cytotoxic T lymphocytes was substantially elevated in the ECLI group compared with the other groups (Fig. 6c, d and SI Fig. 9), suggesting efficient infiltration and activation of T cells within the tumor. Additionally, the proportion of CD45^+^CD49b^+^ NK cells significantly higher in the ECLI group was than in the other groups (Fig. 6e and f), indicating potent activation of NK cell-mediated antitumor immunity. To verify that ECLI activates the immune system, we analyzed tumor-draining lymph nodes. The results showed a significant increase in CD8^+^ T cells in the draining lymph nodes (SI Fig. 10). Furthermore, the percentage of F4/80^+^CD86^+^ M1-polarized macrophages in the ECLI group was notably higher than in the PBS, EC, and LIGHT groups (Fig. 6g and h), demonstrating ECLI-induced skewing of macrophages toward a proinflammatory phenotype. In addition, levels of IFN-γ, TNF-α, IL-1β, and granzyme B were all significantly higher in the ECLI group than in the other groups (Fig. 6i–l), further confirming robust activation of effector immune pathways. In summary, ECLI orchestrates synergistic activation of APCs, T cells, NK cells, and macrophages while promoting the secretion of proinflammatory cytokines and cytotoxic molecules, thereby achieving profound remodeling of the tumor immune microenvironment and providing immunological mechanistic support for its remarkable antitumor efficacy. These results collectively demonstrate that ECLI treatment profoundly remodels the tumor immune microenvironment and potently activates antitumor immune responses across multiple dimensions.

### 3.7. ECLI markedly reduces 4T1 lung metastasis

**Fig. 7:**
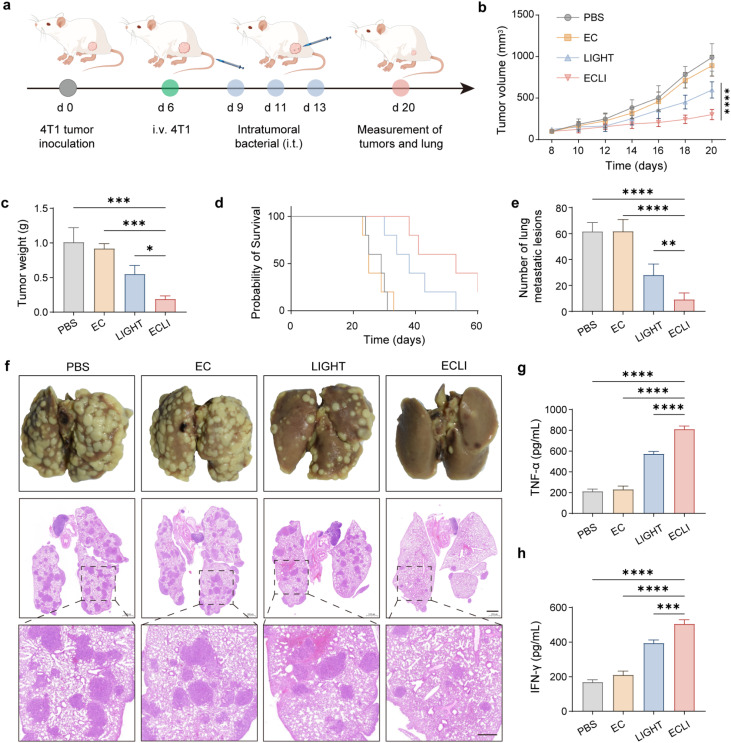
ECLI activates systemic immunity in 4T1 bearing mice. (a) Schematic of the experimental procedure: 4T1 tumor cells were inoculated on day 0, followed by intravenous injection of 4T1 cells on day 6 to induce metastasis, intratumoral bacterial administration on days 9, 11, and 13, and assessments on day 20. (b) Tumor volume dynamics of mice in the PBS, EcN, ECLI, and LIGHT groups over time. (c) Quantification of tumor weight on day 20. (d) Kaplan–Meier survival curves showing the survival probability of mice in different groups. (e) Number of lung metastatic nodules in each group. (f) Gross images (upper panels) and H&E-stained histology (middle and lower panels) of lungs, depicting metastatic lesions; scale bar: 100 µm (middle panels). (g and h) Serum levels of TNF-α (g) and IFN-γ (h) measured by ELISA. Data are presented as mean values ± SEM (*n* = 5 biologically independent samples for (b)–(e) and (g)–(h)).

Given the pronounced metastatic nature of TNBC, we next assessed whether EcN-LIGHT therapy can suppress distant metastasis. As illustrated in the experimental timeline (Fig. 7a), 4T1 tumor cells were inoculated into mice on day 0, followed by intravenous injection of 4T1 cells on day 6 to induce experimental lung metastasis, a critical feature of this model. Intratumoral bacterial administration was then performed on days 9, 11, and 13, with comprehensive assessments conducted on day 20. Tumor volume kinetics (Fig. 7b) revealed that the ECLI group exhibited significantly slower tumor growth compared with the PBS, EcN, and LIGHT groups. Consistent with this, tumor weight was drastically reduced in the ECLI group (Fig. 7c). Survival analysis (Fig. 7d) showed that ECLI treatment prolonged overall survival, with mice in this group surviving longer than those in the other groups. In terms of metastasis, the number of lung metastatic nodules was markedly decreased in the ECLI group (Fig. 7e), and histological examination (Fig. 7f) confirmed fewer and smaller metastatic lesions in the lungs of ECLI-treated mice. Additionally, systemic immune activation was evident, as ECLI treatment significantly elevated the levels of proinflammatory cytokines, TNF-α (Fig. 7g) and IFN-γ (Fig. 7h) in serum, indicating robust activation of antitumor immune responses. Collectively, these findings illustrate that ECLI exerts a multifaceted therapeutic effect in the 4T1 model: it potently inhibits primary tumor growth, suppresses metastatic dissemination to the lungs, prolongs survival, and activates systemic antitumor immune responses.

## 4. Conclusion

Our study systematically demonstrated that ECLI exerts potent antitumor efficacy through multidimensional mechanisms in 4T1 breast cancer models. Specifically, ECLI not only effectively inhibits the growth of subcutaneous tumor and reduces the metastasis of tumor foci, but also significantly prolongs the survival period of mice. Moreover, this treatment approach has no obvious related toxic effects. Mechanistically, ECLI profoundly remodeled the tumor immune microenvironment by activating CD80^+^CD86^+^ antigen-presenting cells, promoting the infiltration of CD3^+^CD8^+^ cytotoxic T lymphocytes, expanding CD49b^+^ natural killer cell populations, polarizing F4/80^+^CD86^+^ macrophages toward the M1 proinflammatory phenotype, and upregulating the secretion of key proinflammatory cytokines (IFN-γ, TNF-α, and IL-1β) and the cytotoxic molecule granzyme B, thereby orchestrating a robust antitumor immune response. Compared with the PBS group and groups treated with EcN or LIGHT alone, ECLI exhibited superior therapeutic efficacy and immune activation efficiency, emphasize its potential as a new strategy for cancer immunotherapy. Further investigations with larger sample sizes, optimized administration regimens, and combination therapy modalities are warranted to explore its clinical translation prospects.

## Author contributions

XY. L. and SJ. D. contributed equally to this work. XY. L. and B. Y. performed the bacterial engineering, molecular construction, and *in vitro* functional assays. SJ. D. and B. Y. conducted the animal experiments and data analysis. SJ. D. assisted with plasmid preparation and protein characterization. Y. W. contributed to cytokine detection and immunofluorescence analysis. W. X. conceived and supervised the project, analyzed the data, and revised the manuscript. All authors discussed the results and approved the final version of the manuscript.

## Conflicts of interest

The authors declare that there is no potential conflicts of interest and no commercial or financial relationships in this study.

## Supplementary Material

RA-016-D5RA09644H-s001

## Data Availability

The data supporting this article have been included as part of the supplementary information (SI). Supplementary information: plasmid profile (Fig. S1), bacterial growth curve OD600 (Fig. S2), as well as main tissue HE staining (Fig. S3), serum biochemical indicators (Fig. S4), blood routine test (Fig. S5), and mouse weight changes (Fig. S6). There were also serum and tumor tissue Flag label detection (Fig. S7), flow cytometry gate logic diagrams (Fig. S8 and S9), as well as CD8 T cells in the tumor-draining lymph nodes (Fig. S10). See DOI: https://doi.org/10.1039/d5ra09644h.
