# Supplementary material for: Inducible secretion of LIGHT by engineered probiotics enables localized cytokine therapy and robust antitumor immunity
Source: RSC Adv. 2026 May 18;16(21):19511–23. doi: 10.1039/d5ra09644h (PMC13181316; doi:10.1039/d5ra09644h)
Supplement: RA-016-D5RA09644H-s001 [file RA-016-D5RA09644H-s001.pdf]

Supplementary Information (SI) for RSC Advances.
This journal is © The Royal Society of Chemistry 2026

Inducible Secretion of LIGHT by Engineered Probiotics Achieves Localized Cytokine Therapy and Robust Antitumor Immunity

Xinyu Li[1, 2, 4], Shuaijie Ding[2, 4], Biao Yang[2], Yi Wang[3], Wei Xie[2, *]

[1] School of Food and Nutrition, Joint Research Center for Food Nutrition and Health of IHM, Anhui Agricultural University, Hefei, Anhui, 230036, China.

[2] Institute of Health and Medicine (IHM), Hefei Comprehensive National Science Center, Hefei, Anhui, 230000, China.

[3] Department of Immunology, School of Basic Medical Sciences, Center for Big Data and Population Health of IHM, Anhui Medical University, Hefei, Anhui, 230032, China

[4] These authors contributed equally: Xinyu Li, Shuaijie Ding

* e-mail: xiew@ihm.ac.cn

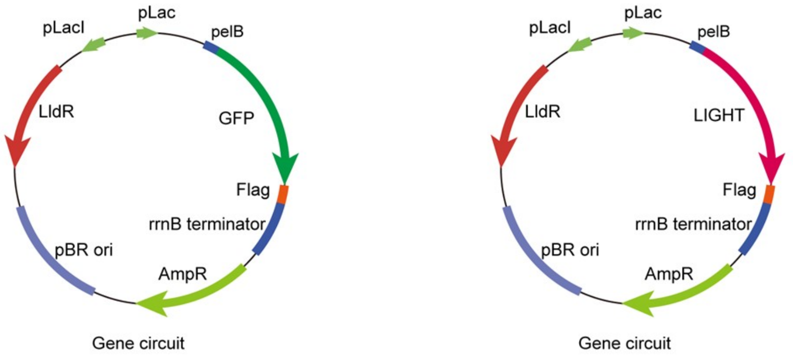

Supplementary Fig. 1. The expression map of GFP or LIGHT in the engineered bacterial plasmid responding to lactic acid

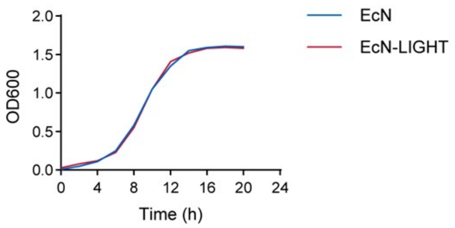

Supplementary Fig. 2. The bacterial growth curve was determined by continuously monitoring OD600 after transferring and not transferring the LIGHT plasmid.

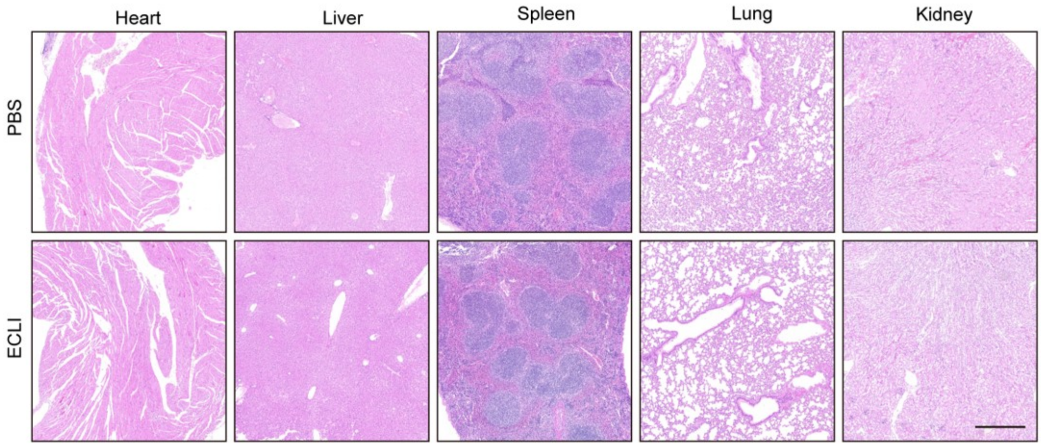

Supplementary Fig. 3. Representative H&E staining of heart, liver, spleen, lung, and kidney from

PBS and ECLI mice. No pathological alterations or inflammatory infiltrates were observed, Scale bar=100 μm.

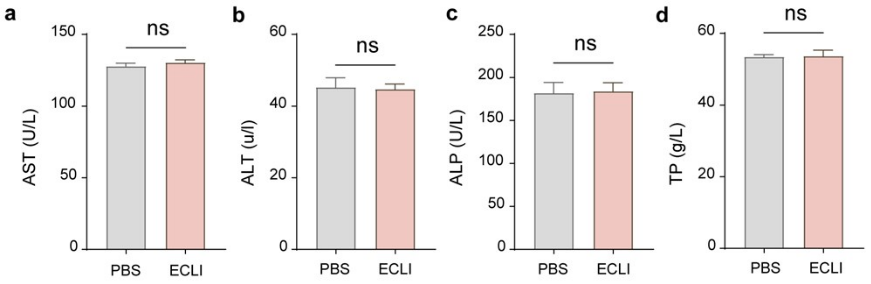

Supplementary Fig. 4. (a-d) Serum biochemical indices showing comparable AST, ALT, ALP, and TP levels between groups, indicating preserved liver function. Data represent mean ± SEM, *n* = 3 mice per group.

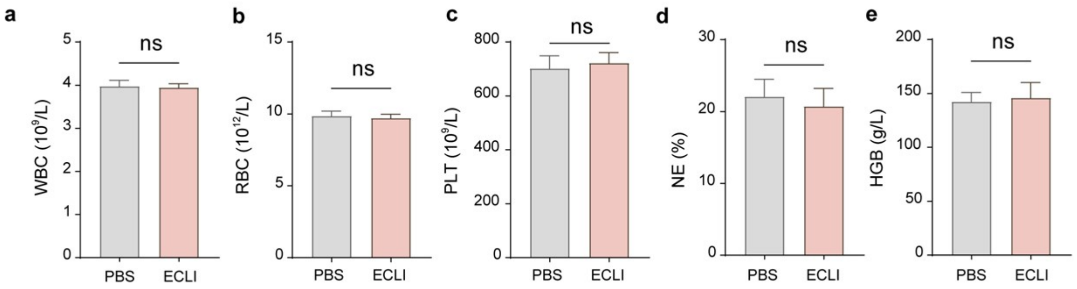

Supplementary Fig. 5. (a-e) Hematological parameters including WBC, RBC, PLT, NE, and HGB counts, all within normal ranges and statistically nonsignificant (*ns*). Data represent mean ± SEM, *n* = 3 mice per group.

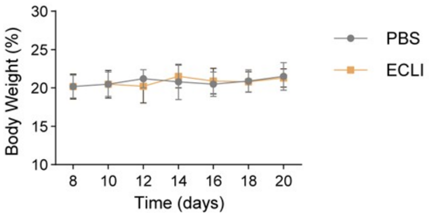

Supplementary Fig. 6. Changes in mouse body weight following different treatments

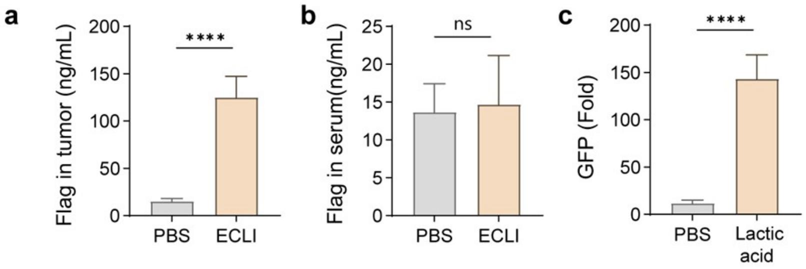

Supplementary Fig. 7. Detection of the Flag tag in tumors and serum (a, b); the Flag tag indicates LIGHT levels. Tumors were isolated and cultured, and expression was induced with lactate (c)

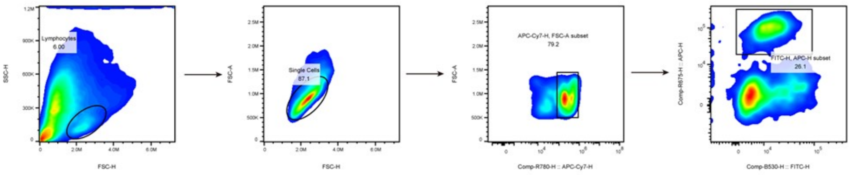

Supplementary Fig. 8. Flow cytometric analysis of $CD45^{+}$ $CD3^{+}CD8^{+}$ cells.

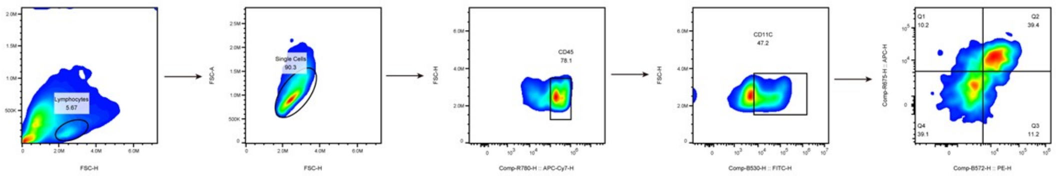

Supplementary Fig. 9. Flow cytometric analysis of $CD80^{+}CD86^{+}$ cells.

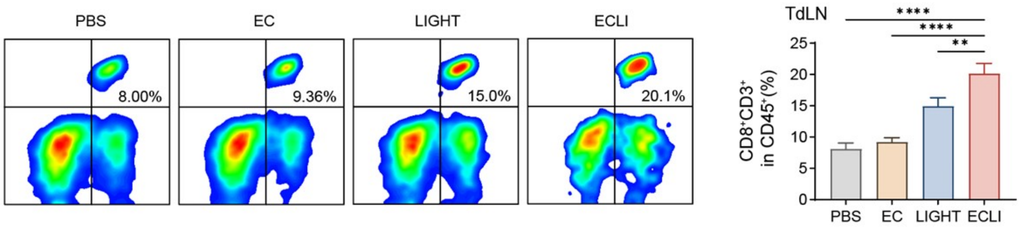

Supplementary Fig. 10. Scatter plots and statistical graphs of CD8+ T cells in tumor-draining lymph nodes obtained via flow cytometry
